# 3D Printing of PLLA/Biomineral Composite Bone Tissue Engineering Scaffolds

**DOI:** 10.3390/ma15124280

**Published:** 2022-06-17

**Authors:** Fangli Gang, Weilong Ye, Chunyang Ma, Wenting Wang, Yi Xiao, Chang Liu, Xiaodan Sun

**Affiliations:** 1 Department of Biology, Xinzhou Teachers University, Xinzhou 034000, China; wangwtsci@163.com (W.W.); xiao1f@163.com (Y.X.); 2 Beijing Key Laboratory of Tooth Regeneration and Function Reconstruction, Beijing Stomatological Hospital, School of Stomatology, Capital Medical University, Beijing 100050, China; yqw83268910@126.com; 3 Key Laboratory of Biomechanics and Mechanobiology, School of Biological Science and Medical Engineering, Beihang University, Beijing 100083, China; 13241214970@163.com; 4 Department of Stomatology, Beijing Friendship Hospital, Capital Medical University, Beijing 100050, China; changliu0406@163.com; 5 Key Laboratory of Advanced Materials of Ministry of Education, School of Materials Science and Engineering, Tsinghua University, Beijing 100084, China

**Keywords:** biominerals, 3D printing, bone tissue engineering

## Abstract

Tissue engineering is one of the most effective ways to treat bone defects in recent years. However, current highly active bone tissue engineering (BTE) scaffolds are mainly based on the addition of active biological components (such as growth factors) to promote bone repair. High cost, easy inactivation and complex regulatory requirements greatly limit their practical applications. In addition, conventional fabrication methods make it difficult to meet the needs of personalized customization for the macroscopic and internal structure of tissue engineering scaffolds. Herein, this paper proposes to select five natural biominerals (eggshell, pearl, turtle shell, degelatinated deer antler and cuttlebone) with widely available sources, low price and potential osteo-inductive activity as functional particles. Subsequently compounding them into L-polylactic acid (PLLA) biomaterial ink to further explore 3D printing processes of the composite scaffold, and reveal their potential as biomimetic 3D scaffolds for bone tissue repair. The research results of this project provide a new idea for the construction of a 3D scaffold with growth-factor-free biomimetic structure, personalized customization ability and osteo-inductive activity.

## 1. Introduction

Bone plays an indispensable role in the human body, and it is required for mechanical support in many human movements. Therefore, either damage or loss of bone tissue can cause great inconvenience to patients, and the treatment of large-area bone defects still faces great challenges. Autologous bone transplantation is the “gold standard” in the field of bone repair, but it is difficult to achieve because of secondary injury to patients [1,2]. Allogeneic bone transplantation also has many deficiencies, such as immune rejection and infectious diseases [3]. Tissue engineering aims to maintain, restore and enhance the function of injured or diseased tissues and organs by developing substitutes [4,5,6]. In recent years, significant progress has been made in tissue engineering, such as the discovery of methods to generate induced pluripotent stem cells [7,8], the finding that substrate stiffness can modulate stem cell differentiation [9], refined delivery mechanisms that enable biochemical cues such as growth factors and cytokines to be presented with improved bioavailability and bioactivity [10,11], increased understanding of the interaction between foreign bodies and the body’s immune surveillance system [12,13], the development of new biomaterials and scaffolds that has led to fabrication of better biomimetic tissues [14,15], and advances in biofabrication technologies including programmed self-assembly and 3D bioprinting [16,17,18,19,20,21,22,23,24,25]. Bone tissue engineering (BTE) puts forward a new scheme to solve this problem, that is, using 3D porous scaffolds to adjust the physiological microenvironment of the defect and induce human bone marrow mesenchymal stem cells (BMSCs) to differentiate into osteoblasts so as to promote the growth of new bone [26,27,28]. Although the addition of active biological components, such as growth factors, can significantly improve the bone tissue repair ability of scaffolds, the high cost, easy inactivation and complex regulatory requirements greatly limit practical application. Therefore, it is an urgent problem for researchers to develop biomimetic 3D scaffolds with growth-factor-free and osteo-inductive activity for bone tissue repair.

Biominerals are functional or pathological crystalline solids existing in organisms. Recently, natural biomaterials have often been utilized for tissue engineering scaffolds [29,30,31,32]. Natural biominerals, such as shells and pearls, have attracted increasing attention in biomedical applications due to their wide availability, low price and superior biophysical properties. The bio-organic macromolecules (polysaccharides, proteins, etc.) and calcium carbonate contained in these natural biominerals may provide a suitable microenvironment for new bone regeneration [33,34,35]. Moreover, the degradation of biominerals releases trace elements, such as zinc, selenium, strontium, calcium, magnesium and phosphorus, which also have potential effects on promoting angiogenesis and osteogenesis. At present, there have been many studies on the mechanisms and applications of biominerals in the field of bone tissue engineering, demonstrating that biominerals have a promising future in the field of bone tissue engineering [36,37]. However, there are still a large number of biological minerals that have not been well studied, and there is a lack of unified and clear comparison between biominerals. Contrasting, classifying and selecting biominerals will be beneficial in advancing the applications of biominerals in bone tissue engineering.

It is generally difficult for biominerals to be directly used in the preparation of scaffolds for BTE because the external shape does not match the defect site. It is a feasible method to grind biominerals into powder and then mix them with polymer materials for the preparation of scaffolds [33,38]. In polymer materials, poly-l-lactide (PLLA) is widely used because of its good biocompatibility [39,40,41]. However, the traditional scaffold preparation methods usually struggle to realize the personalized customization of the macroscopic and internal structure of the scaffold simultaneously [42,43]. The introduction of 3D printing technology has effectively solved this problem. This is in view of the fact that PLLA can be rapidly frozen at −3 °C after dissolving in 1,4-dioxane, and the solvent can volatilize rapidly in vacuum. Therefore, the scaffold was prepared by using the 3D printing method of “room temperature extrusion–freeze receiving-vacuum drying” while1,4-dioxane was selected as the solvent.

**Figure 1:**
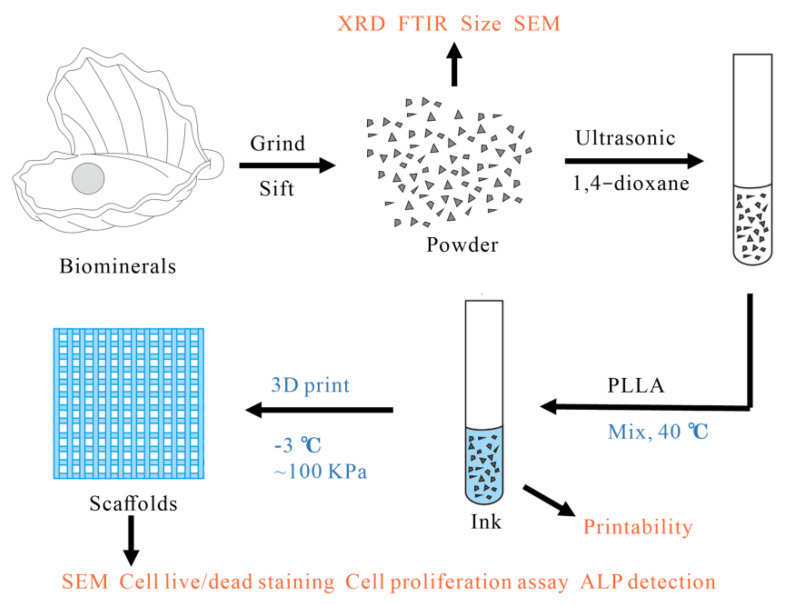
Preparation of scaffold.

In this paper, five common biominerals with potential osteo-induction, including eggshell, pearl, turtle shell, degelatinated deer antler and cuttlebone, were ground into powder, mixed with PLLA, and prepared into scaffolds by 3D printing technology (Figure 1). The physicochemical properties, biocompatibility and osteogenic differentiation ability of the biomineral powders, ink and scaffolds were then tested to evaluate the potential value of these five biominerals for BTE.

## 2. Materials and Methods

### 2.1. Preparation and Characterization of Powder

The 5 kinds of biominerals, eggshell, pearl, turtle shell, degelatinated deer antler and cuttlebone were bought from Beijing Tongrentang. The biominerals were washed clean using deionized water and placed inside a fume hood to dry. A powder mill (Beijing Kewei Yongxing Instrument Co., Ltd., Beijing, China) was used to grind the 5 kinds of biominerals into powder, then a 50 μm sieve was used to screen, and finally collect and was set aside. The 5 kinds of biomineral powder were taken and handed over to the Material Center Laboratory of Tsinghua University for particle size, XRD and FTIR testing. The XRD values were matched with JCPDS values to confirm the mineral composition of the powders. XRD test results were analyzed using MDI jade 6 (Materials Data, Livermore, CA, USA), Omnic V8.2 (Nicolet, Madison, WI, USA) was used to analyze the FTIR test results, and OriginPro 8.5 (OriginLab, Northampton, MA, USA) was used for image rendering.

### 2.2. Preparation and Characterization of Ink

Take 0.15 g eggshell powder, pearl powder, turtle shell powder, degelatinated deer antler powder and cuttlebone powder, respectively, and put them into 10 mL 1,4-dioxane (Sinopharm Chemical Reagent Co., Ltd., Shanghai, China) and use a cell ultrasonic crusher (Ningbo Xinzhi Biotechnology Co., Ltd., Ningbo, China) for 5 min to disperse the powder in 1,4-dioxane. Add 1.5 g PLLA (viscosity 2.4, Beijing Jude Antai Technology Co., Ltd., Beijing, China) and stir at a constant temperature of 40 °C until PLLA is completely dissolved and the powder is evenly dispersed. Pour the prepared ink into the barrel of the 3D printer for standby. Use a 3D printer to print several inks under the pressure of 20, 40, 60, 80, 100, 120, 140 and 160 kPa, respectively, that is, draw a circle with a diameter of 10 mm, and then test the viscosity of the ink and select the appropriate printing pressure by observing its forming effect. The 3D image is drawn by the 3D builder, and the size of the circle is 10 mm × 10 mm × 0.5 mm. A stainless-steel plate was used as the receiver stage for the materials. The inner diameter of the 3D printer nozzle is 410 μm. The temperature is −3 °C.

### 2.3. Preparation of Scaffolds

Draw a 10 mm × 10 mm × 2 mm cuboid 3D image with the 3D builder and import it into the 3D printer control program (Gesim, Radberg, Germany). The 3D printing method of normal temperature extrusion (nozzle diameter 410 μm) and low temperature (−3 °C) reception is used for printing. After printing, put the scaffolds into the freeze dryer for freeze-drying and finalize the shape. After 30 min of low-temperature vacuum drying, the shape of the scaffolds has been basically fixed, but it takes more than 2 h to completely remove 1,4-dioxane. In order to ensure the safety of the scaffolds, place in the fume hood for 7 days.

### 2.4. SEM Observation

The 5 kinds of biomineral powders were individually pasted onto the experimental bench and observed using a scanning electron microscope (S-450 SEM, Hitachi, Tokyo, Japan) after gold spraying. The SEM observation of the scaffolds is similar to this. The prepared scaffolds are cut into small pieces and pasted on the test bench. After spraying gold, the scaffolds can be observed by SEM. The SEM images of scaffolds were analyzed by Image J software, and the information regarding fiber diameter and aperture of different scaffolds was counted.

### 2.5. Cell Live/Dead Staining

Scaffolds of 10 mm × 10 mm × 2 mm in size were placed in a 24-well plate, and pure PLLA was set as control. SD-MSC P5 (purchased from Cyagen Biosciences Inc., Santa Clara, CA, USA) were seeded into the wells at a density of 5 × 10^4^ cells in 500 μL medium per well. After the cells adhered to the wall for 4–6 h, scaffolds were moved into a new 96-well plate. After another 24 h, the medium was aspirated off and washed three times with PBS. Protected from light, calcein/propidium iodide working solution was formulated and replaced the previous medium, and was then incubated at 37 °C incubator for 15 min. The excessive dye was washed twice with PBS, and the well plate was moved to a confocal microscope (Zeiss LSM710, Oberkochen, Germany) for observation. Excitation wavelengths for PI and C-AM are 561 nm and 488 nm, and emission wavelengths for PI and C-AM are 579 nm and 537 nm, respectively. Scanning objective lens of 10× was used in the examination.

### 2.6. Cell Proliferation Assay

Briefly, 6 types of scaffolds were sterilized by ^60^Co irradiation at 5 Mrad for further use. These scaffolds are cut to 5 mm × 5 mm × 2 mm in size and placed in 96-well plate, *n* = 3. SD-MSC P5 was resuspended in complete medium and evenly planted into 96-well plate at the density of 8000 cells in 200 μL medium per well. On 1/3/5 days, 10% *v*/*v* CCK8 working solution was prepared and replaced the previous medium. After incubation in 5% CO_2_ incubator at 37 °C for 2 h, 100 μL suspension per well was moved into a new 96-well plate and absorption was measured by microplate reader at 450 nm. The quantitative data were plotted by Graphpad Prism V7 (GraphPad Software, San Diego, CA, USA) and statistically analyzed by SPSS 19 (IBM, Chicago, IL, USA).

### 2.7. ALP Detection

The preparation of cells and scaffolds is the same as that in 2.7. SD-MSC was seeded to the surface of the scaffold in the 24-well plate at the density of 5 × 10^4^ cells in 500 uL medium per well. After 6 h of cell adhesion, the previous medium was replaced with osteogenic induction medium containing mem-α, 15% fetal bovine serum, 1% penicillin/streptomycin, 1% glutamine, 100 μM/mL of ascorbic acid, 2 mM of β-glycerophosphate, 1.8 mM of KH_2_PO_4_ and 10 nM of dexamethasone. The protein was extracted after 4/6 days of induction, and the protein concentration was measured at 595 nm by Bradford assay (semefeld article number: 23,200, standard curve is available on the official website). ALP concentration was measured at 405 nm according to the manufacturer’s protocol for ALP Activity Kit (Sigma Aldrich, St. Louis, MO, USA). ALP activity = ALP concentration/total protein concentration.

## 3. Results and Discussions

### 3.1. Powder Test

**Figure 2:**
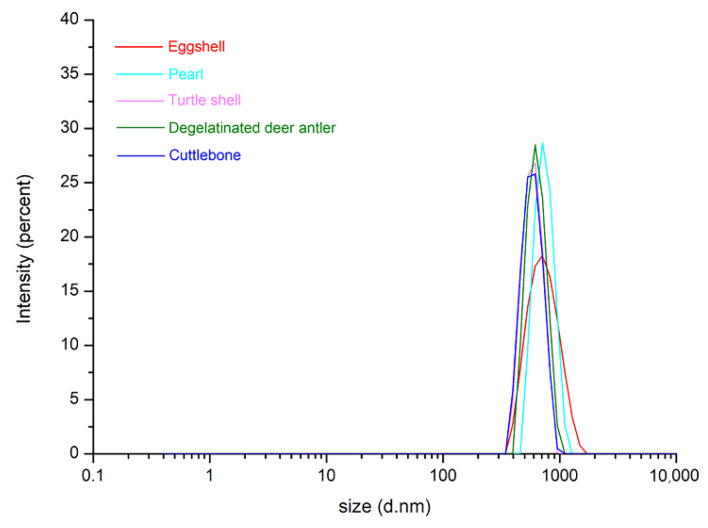
Particle size distribution of eggshell, pearl, turtle shell, degelatinated deer antler and cuttlebone powders.

To gain more detailed insight into the structure and composition of these five biominerals, the particle size, SEM, XRD, and FTIR tests were performed after the preparation for the five kinds of powders had been completed. The particle size distribution test results are shown in Figure 2.

**Figure 3:**
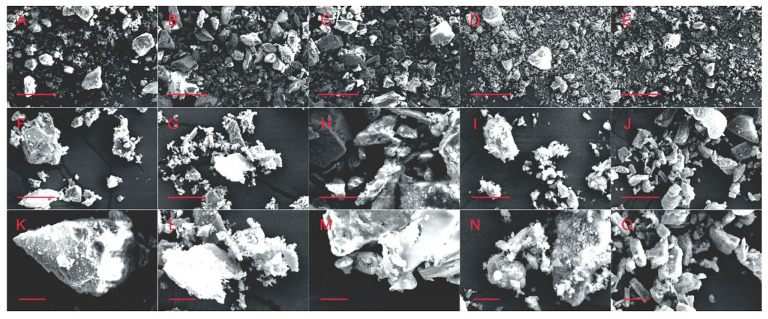
SEM images of eggshell powders (**A**,**F**,**K**), Pearl powders (**B**,**G**,**L**), turtle shell powders (**C**,**H**,**M**), degelatinated deer antler powders (**D**,**I**,**N**) and cuttlebone powders (**E**,**J**,**O**), the scale bar is 300 μm (**A**–**E**)/30 μm (**F**–**J**)/10 μm (**K**–**O**).

Most particle sizes are between 500–900 nm, while the diameter of the 3D printer nozzle is 410 μm, so particles of this size will not block the printer nozzle. However, some large particles are small in number but large in size, which will lead to the risk of nozzle blockage. Therefore, it is necessary to observe the powder via SEM under different magnification. As shown in Figure 3, from the SEM images with different magnifications, it can be seen that the particle size distribution range of the five kinds of biomineral powders is wide, ranging from nano scale to micron scale. Many larger sized particles are present in the powder, but they are all below 300 μm in size, posing a lesser risk of clogging of the nozzle. The observed particle sizes of these five kinds of biomineral powders were generally consistent and did not show large differences. In addition, it can also be seen in the SEM image that there is a certain phenomenon of mutual adsorption between particles, which will cause some trouble for 3D printing. Therefore, it should be fully dispersed in the ink preparation process.

**Figure 4:**
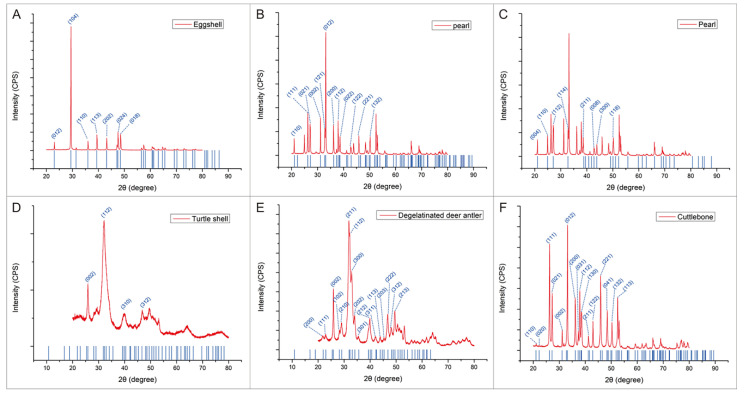
XRD test results of eggshell, pearl, turtle shell, degelatinated deer antler and cuttlebone powder. (**A**) Comparison of XRD results between calcite and eggshell powder. (**B**) Comparison of XRD results between aragonite and pearl powder. (**C**) Comparison of XRD results between vaterite and pearl powder. (**D**) Comparison of XRD results between hydroxylapatite and turtle shell powder. (**E**) Comparison of XRD results between hydroxylapatite and degelatinated deer antler powder. (**F**) Comparison of XRD results between aragonite and cuttlebone powder.

**Figure 5:**
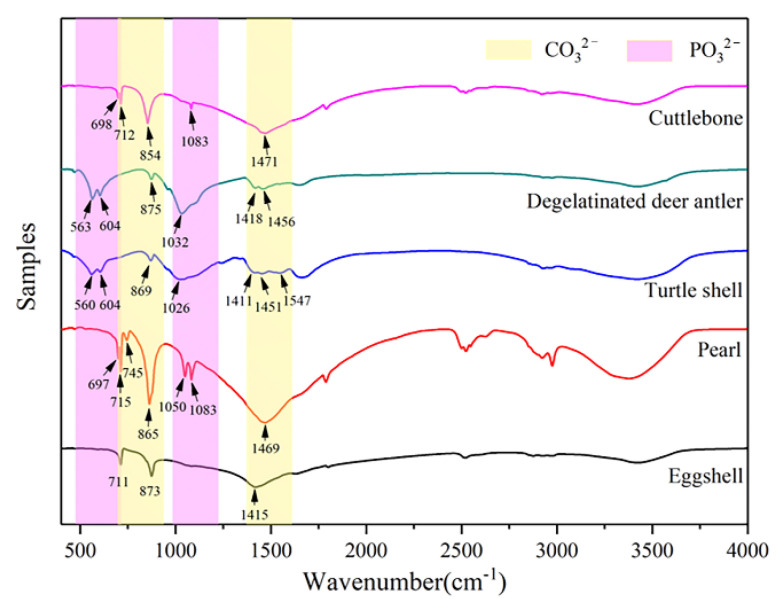
FTIR test results of eggshell, pearl, turtle shell, degelatinated deer antler and cuttlebone powder.

The mineral composition of these five kinds of biominerals can be determined by XRD (Figure 4) and FTIR (Figure 5).

According to the analysis results of XRD and FTIR, the main chemical component of minerals in eggshell, pearl and cuttlebone is CaCO_3_, while the main chemical component of minerals in turtle shell and degelatinated deer antler is Ca_5_(PO_4_)_3_(OH). Among them, the main mineral type in eggshell is calcite; pearls contain two crystal types of CaCO_3_, aragonite and vaterite; the main mineral type in cuttlebone is aragonite; and the main mineral type in turtle shell and degelatinated deer antler is hydroxylapatite. Therefore, through the comparison of these five kinds of biominerals, whether there are differences in the application of biominerals containing different mineral types in the field of BTE can be judged. Chemical composition and particle size of the biominerals are shown in Table 1.

### 3.2. Ink Test

**Figure 6:**
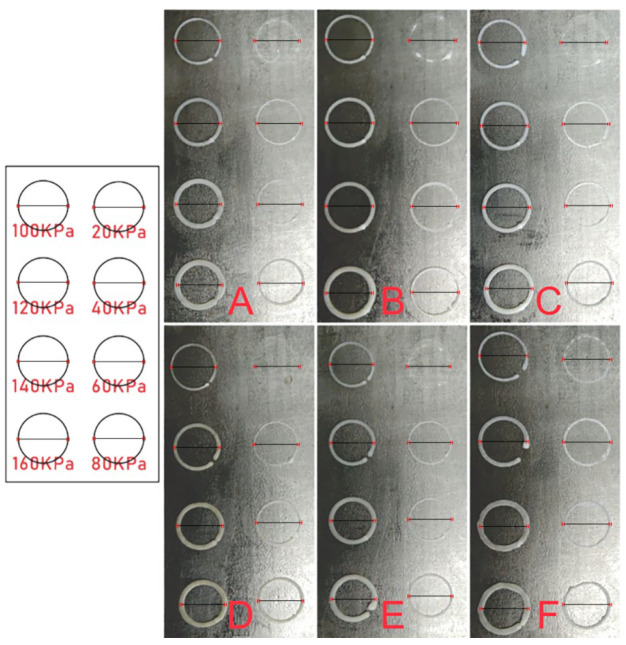
3D printing effect of pure PLLA (**A**), PLLA/eggshell powder (**B**), PLLA/pearl powder (**C**), PLLA/turtle shell powder (**D**), PLLA/degelatinated deer antler powder (**E**), and PLLA/cuttlebone powder (**F**) composite ink under different air pressures (20, 40, 60, 80, 100, 120, 140, 160 kPa).

In the process of 3D printing, the ink is extruded by the nozzle under appropriate air pressure and adhered to the receiving platform or the previous layer of material, and quickly frozen in a low temperature environment. Therefore, the selection of appropriate air pressure plays a vital role in 3D printing. Air pressure that is too low will lead to problems such as difficult extrusion of ink, and air pressure that is too high will lead to problems such as overly thick fiber, deformation and slowing down the speed of freezing. As shown in Figure 6, the printing effects of six kinds of composite inks (pure PLLA, PLLA/eggshell powder, PLLA/pearl powder, PLLA/turtle shell powder, PLLA/degelatinated deer antler powder and PLLA/cuttlebone powder) at 20, 40, 60, 80, 100, 120, 140 and 160 kPa, respectively. Ideally, the 3D printed circle should have a diameter of 1 cm and a fiber width of 410 μm, as indicated by the line segments in the figure. Obviously, with the increase in air pressure, the width of the line gradually increases. Through the comparison of design effects and actual effects, it is possible to verify the printability of this material and find out the suitable air pressure. It can be seen from the figure that the best printing pressure of pure PLLA ink is 100–120 kPa, the best printing pressure of PLLA/eggshell powder composite ink is about 100 kPa, the best printing pressure of PLLA/pearl powder composite ink is 100–120 kPa, the best printing pressure of PLLA/turtle shell powder composite ink is about 120 kPa, and the best printing pressure of PLLA/degelatinated deer antler powder and PLLA/cuttlebone powder composite ink is 100–120 kPa. In a word, the addition of these five kinds of powders has little effect on ink viscosity. It is more appropriate to select the air pressure of 100 ± 20 kPa according to the actual situation in the printing process.

Furthermore, according to the size distribution of particles, it can be judged that nozzles with an inner diameter above 300 μm are not easily clogged and nozzles with an inner diameter below 300 μm may also be used, but the risk of clogging will increase and lead to a decrease in the success rate of scaffold preparation. Nozzle clogging with a larger inner diameter carries little risk, but the increased diameter slows the freezing rate which in turn causes deformation of the scaffolds. Reducing the ambient temperature can accelerate the rate of freeze forming and reduce the risk of deformation. If the receiving base plate is used to cool down rather than the overall environment, the temperature will increase as it builds away from the base plate. For the macro size of the support, when the base plate temperature is −3 °C, the scaffolds can be printed to about 4 mm at most, and the length and width of the scaffolds are determined by the size of the receiving plate. If the whole printing environment is cooled down, the maximum size is determined by the size of the machine’s printing space. The ink mixed with biomineral powders is more likely to be cryo-shaped during printing, possibly related to the presence of powders.

### 3.3. SEM Observation of the Scaffolds

**Figure 7:**
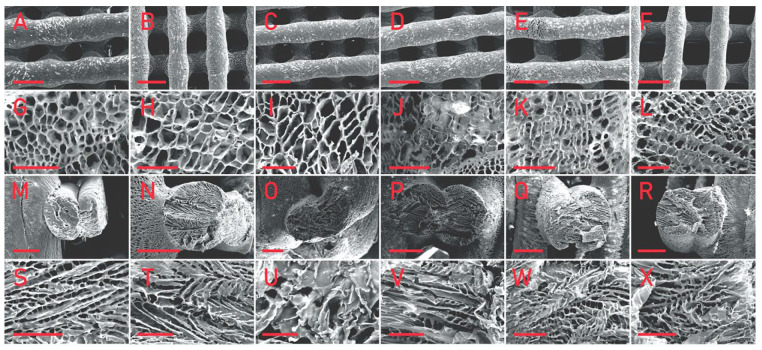
SEM images of pure PLLA (**A**,**G**,**M**,**S**), PLLA/eggshell powder (**B**,**H**,**N**,**T**), PLLA/pearl powder (**C**,**I**,**O**,**U**), PLLA/turtle shell powder (**D**,**J**,**P**,**V**), PLLA/degelatinated deer antler powder (**E**,**K**,**Q**,**W**), and PLLA/cuttlebone powder (**F**,**L**,**R**,**X**) composite scaffolds. (**A**–**F**) shows the macro morphology of the front of the scaffolds, the scale bar is 500 μm; (**G**–**L**) shows the micro morphology of the front of the scaffolds, the scale bar is 50 μm; (**M**–**R**) shows the macro morphology of the cross section of the scaffolds, the scale bar is 200 μm; and (**S**–**X**) shows the micro morphology of the cross section of the scaffolds, the scale bar is 50 μm.

SEM observation was carried out on the prepared 3D printing porous scaffolds, as shown in Figure 7.

**Figure 8:**
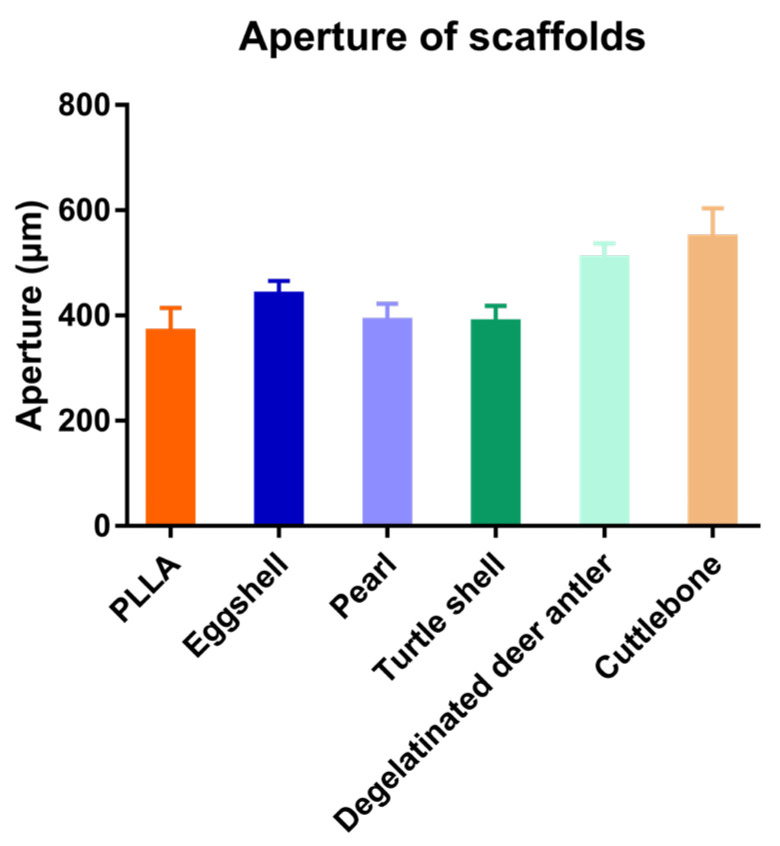
Aperture of the five kinds of scaffolds.

**Figure 9:**
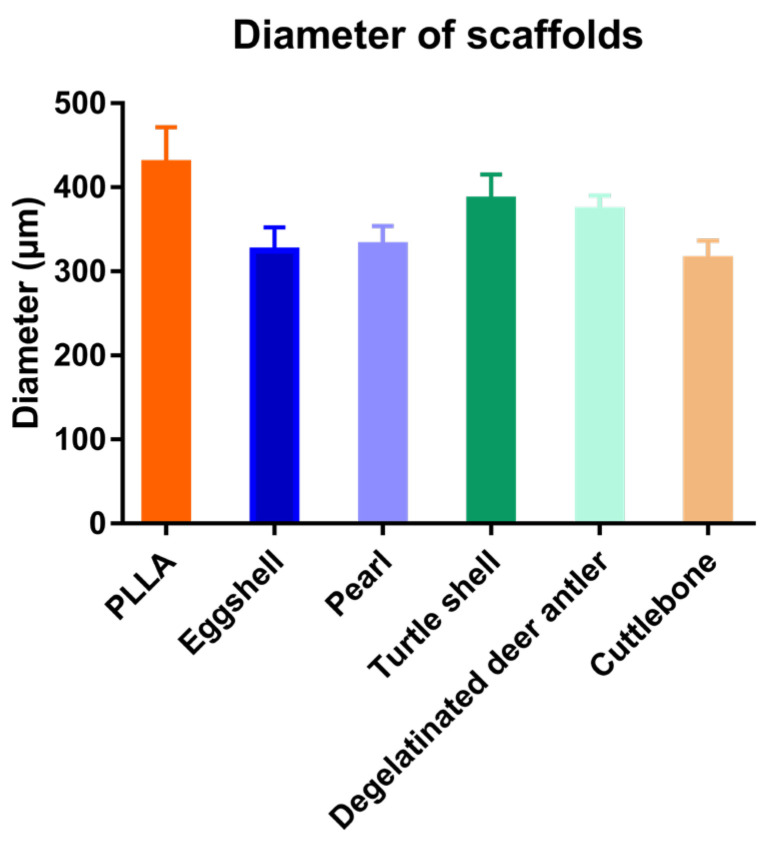
Fiber diameters of the five kinds of scaffolds.

The scaffolds mixed with various biomineral powders have almost the same pore structure as pure PLLA scaffolds, that is, they have both micron and nano pores. This pore structure is one of the advantages of 3D printing porous scaffolds. As shown in the figure, the micro pores of the scaffolds can reach about 400 μm, which is conducive to cell migration, while the nano pores distributed on the surface of the scaffold increase the surface area of the scaffold, which is conducive to cell adhesion and growth. It can be seen in the cross-sectional view that nano pores fill the whole scaffold, and the morphology of nano pores distributed in the scaffold is almost the same as that on the surface of the scaffold. The 3D printing process conducted at low temperature is affected by many factors, which have a great impact on its printing effect, and it is very difficult to control its diameter stability. Image J was used to analyze the aperture (Figure 8) and fiber diameter (Figure 9) of the SEM images of the scaffolds. In contrast, the scaffolds mixed with biomineral powder showed certain shrinkages, and the fiber diameter was slightly smaller than that of the control group. On the whole, the difference in the diameter of each group of fibers is mostly less than 100 μm, which is already an ideal state for scaffolds printed by the low-temperature condensation method.

Research on the use of biomineral powders for 3D printing after mixing them with polymeric materials has been extensive, and the means of 3D printing used are diverse. Scaffolds printed using the high-temperature melt method tend to have better molding results with straight fibers, smooth surfaces and fewer pores, and the actual effect is more similar to the design effect [35]. The scaffolds fabricated by the printing method used in this paper were less effective in molding than those fabricated by the high-temperature melting method, but the pores formed by lyophilization made the surface of scaffolds rough and the pore structure was more conducive to cell adhesion and growth, it also had a better promotion effect on bone regeneration. The scaffolds mixed with biomineral powder did not show much difference in printability, scaffold morphology or pore structure compared to PLLA.

### 3.4. Cell Experiment

**Figure 10:**
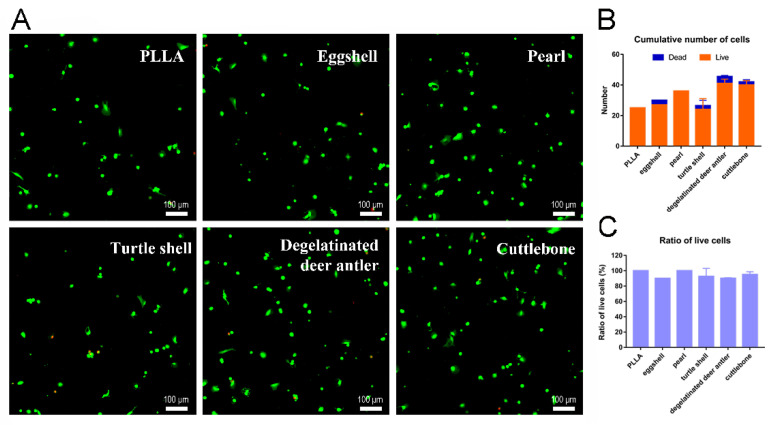
(**A**) Live/dead staining images of cells on pure PLLA, PLLA/eggshell powder, PLLA/pearl powder, PLLA/turtle shell powder, PLLA/degelatinated deer antler powder and PLLA/cuttlebone powder composite scaffolds. (**B**) Cumulative quantification of live/dead cells. (**C**) Ratio of live/dead cells.

As shown in Figure 10, after 24 h of cell adhesion, the cells on the surface of the well plate are evenly distributed. The fluorescence test results showed that, in the sustained-release solution environment of each group of scaffolds, the cells were typical green living cells, while the red dead cells were basically invisible. The observation of cells on scaffolds in each group showed that the number of cells was relatively small, but most of them were still living cells. Most of these cells are distributed along the axial direction of the intersecting scaffold fibers, and a small number of cells will also gather in the pores of the scaffold. PLLA scaffolds are hydrophobic, so less cells remain on the fibers in the early stage of adhesion, and the long-term effect of cell proliferation needs to be verified by CCK8 experiments. However, the staining results showed that the cell state was good enough to confirm the excellent biosafety of the scaffold. As we can see in Figure 10B,C, >90% of cells at scaffolds were live on all groups, which indicated the satisfying biosafety of our PLLA and PLLA/biomineral scaffolds.

**Figure 11:**
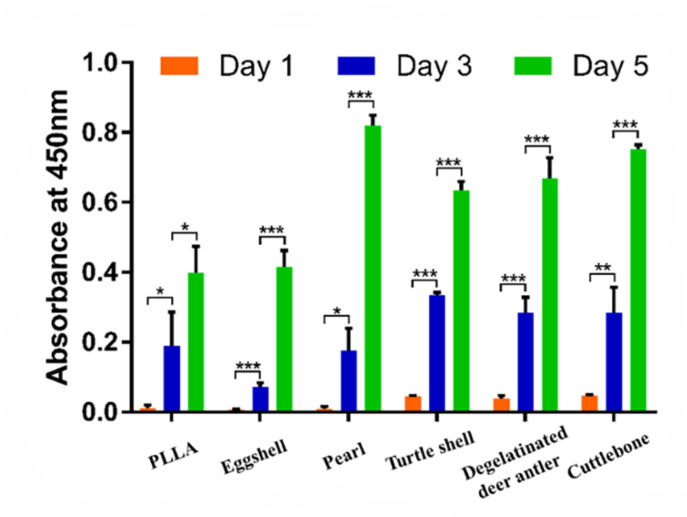
Biocompatibility test of pure PLLA, PLLA/eggshell powder, PLLA/pearl powder, PLLA/turtle shell powder, PLLA/degelatinated deer antler powder and PLLA/cuttlebone powder scaffolds. (* *p* < 0.05, ** *p* < 0.01, *** *p* < 0.001).

As shown in Figure 11, the cells on all groups of scaffolds showed a typical growth trend on the 1st/3rd/5th day after seeding. From the 1st day to the 3rd day, the OD values of the control group (pure PLLA), pearl group and cuttlebone group increased significantly (*p* < 0.05), while the eggshell group, turtle shell group and degelatinated deer antler group had very significant statistical significance (*p* < 0.001). From the 3rd day to the 5th day, the increase of OD value in the PLLA group was statistically significant (*p* < 0.05), while other groups showed very significant statistical significance (*p* < 0.001). In the pure PLLA, eggshell and pearl groups, the OD values on the first day were 0.011 ± 0.009, 0.007 ± 0.002 and 0.008 ± 0.008, respectively, which were lower than the 0.045 ± 0.003, 0.04 ± 0.007 and 0.047 ± 0.002 in the turtle shell group, degelatinated deer antler group and cuttlebone group. On the 5th day, the PLLA group (0.4 ± 0.075) and eggshell group (0.415 ± 0.046) were still lower than the pearl group (0.82 ± 0.03), degelatinated deer antler group (0.668 ± 0.059) and cuttlebone group (0.753 ± 0.012). At the same time, the OD value of the turtle shell group also increased to 0.634 ± 0.026. From the 1st day to the 5th day, the OD values of the turtle shell group, degelatinated deer antler group and cuttlebone group remained relatively high.

**Figure 12:**
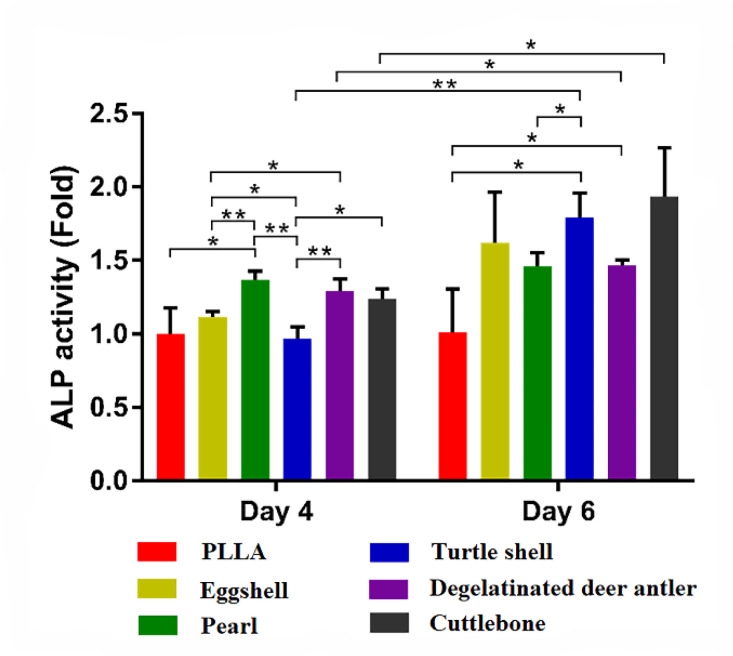
ALP activity test of pure PLLA, PLLA/eggshell powder, PLLA/pearl powder, PLLA/turtle shell powder, PLLA/degelatinated deer antler powder and PLLA/cuttlebone powder scaffolds. (* *p* < 0.05, ** *p* < 0.01).

In order to evaluate the effect of drug loaded scaffolds on osteogenic differentiation, the early index of osteogenesis, ALP activity, was measured. As shown in Figure 12, the ALP activity of PLLA on the 4th day was set as the control. From the 4th day to the 6th day of induction, the ALP activity of each group increased, and the OD of the turtle shell group, degelatinated deer antler group and cuttlebone group increased significantly (*p* < 0.05). On the 4th day, the ALP activity of the pearl group, degelatinated deer antler group and cuttlebone group were higher than that of PLLA group, eggshell group and turtle shell group. However, on the 6th day, the eggshell group and turtle shell group increased rapidly, which was higher than that of the pearl group. On the 4th and 6th day, the cuttlebone group was always the group with higher ALP activity. The stronger trends of osteogenic differentiation indicated higher potential of bone matrix secretion after implantation in vivo.

After the cells were seeded on the scaffold, cells adhesion for 4~6 h in the first 24 h was crucial to their viability, so live/dead staining was performed on the first day. Due to its hydrophobicity, PLLA was usually queried for its bioactivity of osteogenic properties, such as cell migration, adhesion, proliferation and differentiation [44,45,46,47]. It was reported to be applicable as osteo-conductive but not osteo-inductive material [48,49]. Various modifications have been used for better cell behaviors on PLLA scaffolds, such as microscopic shaping, electrospinning, molding, and 3D printing [46,47,50,51]. Another direct functionalization is introducing other bioactive materials such as collagen, biominerals and growth factors [52,53,54]. Considering the stability and economic cost of involved materials, biominerals were applied here for better osteogenic induction and cell behaviors. Eggshell and pearls are common bioactive materials, but they only provide osteo-conductive function rather than osteo-inductive [55]. However, turtle shell, degelatinated deer antler and cuttlebone are traditional biomaterials [56,57,58,59]. Among the scaffold preparation methods, 3D printing is a promising strategy for bioactivity involvement, as well as strong customizability according to the specific morphology of in situ defect areas [60]. Similarly with previous publications, cells poorly adhered on PLLA scaffolds, but stayed well on the PLLA/biominerals scaffolds. The significantly improved ALP activities of SD-MSC showed exciting potential for bone matrix secretion and in vivo implantation. The results provide great evidence for further in vivo study, which would do great favor for bone defect repair in translational medicine. Some studies have shown that the organic matrix contained in biological minerals can promote the differentiation of bone marrow mesenchymal stem cells, but cell proliferation and differentiation have no obvious relationship with the molecular weight of organic matrices. For example, YS Liu [33,36] divided a pearl water soluble organic matrix into an organic matrix < 1 kDa and organic matrix > 3.5 kDa according to molecular weight, and compared the effects of an organic matrix with different molecular weights of aragonite and spheroidal aragonite on mouse MC3T3-E1 cells and human bone marrow mesenchymal stem cells. The results showed that the water-soluble organic matrix had no promoting effect on the proliferation of the two types of cells, but was able to promote the differentiation of the two types of cells into an osteogenic direction. Both the composition and structure of natural biominerals are more complex than those of artificial materials, so it is necessary in future studies to decompose their constituents to investigate the mechanism of action of their different constituents on cells, which will help to distinguish and refine more efficient fractions in biominerals to better promote the application of biominerals in the field of tissue engineering. At present, the mechanisms and applications of biominerals in the field of bone tissue engineering have been widely studied, and many other biominerals, except for the five kinds of biominerals studied in this paper, have been intensively studied, such as biosilica isolated from marine sponges et al. [61,62,63,64]. Studies have proven that biosilica isolated from marine sponges can function to balance pH, control calcium absorption/release and enhance cell viability/proliferation [65,66]. Similar to that, a large number of biominerals have now been shown to have the ability to promote cell proliferation and differentiation, which holds great promise in tissue engineering. In future studies, it is necessary to deeply investigate and contrast the commonalities and differences of biominerals on cell proliferation and differentiation and explore their effects under different usage scenarios to better advance their applications in the field of tissue engineering.

## 4. Conclusions

The main components of eggshell, pearl, turtle shell, degelatinated deer antler and cuttlebone are organic matter and calcium carbonate and/or calcium phosphate. These five kinds of biominerals all enable the fabrication of 3D porous scaffolds through the process of grinding, screening, compounding with PLLA to prepare 3D printing ink, and subsequent 3D printing and freeze-drying. These five kinds of 3D composite scaffolds all simultaneously possess macro and microscopic multi-stage pores, which are beneficial to cell migration, growth and adhesion. Cell experiments demonstrated the five biomineral composite scaffolds have good biocompatibility and the ability to promote bone regeneration. Therefore, biominerals have a promising future in the design and application of scaffolds for BTE.

## Figures and Tables

**Table 1:** Chemical composition and particle size of the biominerals.

Biomineral	Chemical Composition	Particle Size
Eggshell	CaCO_3_	400–1200 nm
Pearl	CaCO_3_	350–1100 nm
Turtle shell	Ca_5_(PO_4_)_3_(OH)	260–610 nm
Degelatinated deer antler	Ca_5_(PO_4_)_3_(OH)	180–6500 nm
Cuttle bone	CaCO_3_	300–610 nm

## Data Availability

Not applicable.

## References

[1] Amini Z., Lari R. (2021). A systematic review of decellularized allograft and xenograft–derived scaffolds in bone tissue regeneration. Tissue Cell.

[2] Baptista R., Guedes M. (2021). Morphological and mechanical characterization of 3D printed PLA scaffolds with controlled porosity for trabecular bone tissue replacement. Mat. Sci. Eng. C.

[3] Su X., Wang T., Guo S. (2021). Applications of 3D printed bone tissue engineering scaffolds in the stem cell field. Regen. Ther..

[4] Langer R., Vacanti J.P. (1993). Tissue engineering. Science.

[5] Khademhosseini A., Vacanti J.P., Langer R. (2009). Progress in tissue engineering. Sci. Am..

[6] Khademhosseini A., Langer R. (2016). A decade of progress in tissue engineering. Nat. Protoc..

[7] Okita K., Ichisaka T., Yamanaka S. (2007). Generation of germline-competent induced pluripotent stem cells. Nature.

[8] Yu J., Vodyanik M.A., Smuga-Otto K., Antosiewicz-Bourget J., Frane J.L., Tian S., Nie J., Jonsdottir G.A., Ruotti V., Stewart R. (2007). Induced pluripotent stem cell lines derived from human somatic cells. Science.

[9] Engler A.J., Sen S., Sweeney H.L., Discher D.E. (2006). Matrix elasticity directs stem cell lineage specification. Cell.

[10] Martino M.M., Briquez P.S., Ranga A., Lutolf M.P., Hubbell J.A. (2013). Heparinbinding domain of fibrin (ogen) binds growth factors and promotes tissue repair when incorporated within a synthetic matrix. Proc. Natl. Acad. Sci. USA.

[11] Pakulska M.M., Miersch S., Shoichet M.S. (2016). Designer protein delivery: From natural to engineered affinity-controlled release systems. Science.

[12] Veiseh O., Doloff J.C., Ma M., Vegas A.J., Tam H.H., Bader A.R., Li J., Langan E., Wyckoff J., Loo W.S. (2015). Size- and shape-dependent foreign body immune response to materials implanted in rodents and non-human primates. Nat. Mater..

[13] Vegas A.J., Veiseh O., Doloff J.C., Ma M., Tam H.H., Bratlie K., Li J., Bader A.R., Langan E., Olejnik K. (2016). Combinatorial hydrogel library enables identification of materials that mitigate the foreign body response in primates. Nat. Biotechnol..

[14] Langer R., Vacanti J.P., Vacanti C.A., Atala A., Freed L.E., Vunjak-Novakovic G. (1995). Tissue engineering: Biomedical applications. Tissue Eng..

[15] Vacanti J.P., Langer R. (1999). Tissue engineering: The design and fabrication of living replacement devices for surgical reconstruction and transplantation. Lancet.

[16] Qi H., Ghodousi M., Du Y., Grun C., Bae H., Yin P., Khademhosseini A. (2013). DNA-directed self-assembly of shape-controlled hydrogels. Nat. Commun..

[17] Todhunter M.E., Jee N.Y., Hughes A.J., Coyle M.C., Cerchiari A., Farlow J., Garbe J.C., LaBarge M.A., Desai T.A., Gartner Z.J. (2015). Programmed synthesis of three-dimensional tissues. Nat. Methods.

[18] Cohen D.L., Malone E., Lipson H., Bonassar L.J. (2006). Direct freeform fabrication of seeded hydrogels in arbitrary geometries. Tissue Eng..

[19] Khalil S., Nam J., Sun W. (2005). Multi-nozzle deposition for construction of 3d biopolymer tissue scaffolds. Rapid Prototyp. J..

[20] Miller J.S., Stevens K.R., Yang M.T., Baker B.M., Nguyen D.-H.T., Cohen D.M., Toro E., Chen A.A., Galie P.A., Yu X. (2012). Rapid casting of patterned vascular networks for perfusable engineered three-dimensional tissues. Nat. Mater..

[21] Kolesky D.B., Truby R.L., Gladman A.S., Busbee T.A., Homan K.A., Lewis J.A. (2014). 3D bioprinting of vascularized, heterogeneous cell-laden tissue constructs. Adv. Mater..

[22] Murphy S.V., Atala A. (2014). 3D bioprinting of tissues and organs. Nat. Biotechnol..

[23] Colosi C., Shin S.R., VManoharan V., Massa S., Costantini M., Barbetta A., Dokmeci M.R., Dentini M., Khademhosseini A. (2016). Microfluidic bioprinting of heterogeneous 3D tissue constructs using low-viscosity bioink. Adv. Mater..

[24] Ober T.J., Foresti D., Lewis J.A. (2015). Active mixing of complex fluids at the microscale. Proc. Natl. Acad. Sci. USA.

[25] Kang H.-W., Lee S.J., Ko I.K., Kengla C., Yoo J.J., Atala A. (2016). A 3D bioprinting system to produce human-scale tissue constructs with structural integrity. Nat. Biotechnol..

[26] Kim D., Lee S., Kim M., Jeong Y., Lee S. (2021). Exosome-coated silk fibroin 3D-scaffold for inducing osteogenic differentiation of bone marrow derived mesenchymal stem cells. Chem. Eng. J..

[27] Wang M., Li H., Yang Y., Yuan K., Zhou F., Liu H., Zhou Q., Yang S., Tang T. (2021). A 3D-bioprinted scaffold with doxycycline-controlled BMP2-expressing cells for inducing bone regeneration and inhibiting bacterial infection. Bioact. Mater..

[28] Ou M., Huang X. (2021). Influence of bone formation by composite scaffolds with different proportions of hydroxyapatite and collagen. Dent. Mater..

[29] Li N., Guo X., Tang X., Xing Y., Pang H. (2022). Three-dimensional Co_2_V_2_O_7_·nH_2_O superstructures assembled by nanosheets for electrochemical energy storage. Chin. Chem. Lett..

[30] Su X., Xian C., Gao M., Liu G., Wu J. (2021). Edible materials in tissue regeneration. Macromol. Biosci..

[31] Huang K., Liu G., Gu Z., Wu J. (2020). Tofu as excellent scaffolds for potential bone regeneration. Chin. Chem. Lett..

[32] Huang K., Gu Z., Wu J. (2020). Tofu-Incorporated Hydrogels for Potential Bone Regeneration. ACS Biomater. Sci. Eng..

[33] Liu Y., Huang Q., Feng Q. (2013). 3D scaffold of PLLA/pearl and PLLA/nacre powder for bone regeneration. Biomed. Mater..

[34] Shen Y., Zhu J., Zhang H., Zhao F. (2006). In vitro osteogenetic activity of pearl. Biomaterials.

[35] Du X., Yu B., Pei P., Ding H., Yu B., Zhu Y. (2018). 3D printing of pearl/CaSO_4_ composite scaffolds for bone regeneration. J. Mater. Chem. B.

[36] Huang Q., Liu Y., Ouyang Z., Feng Q. (2020). Comparing the regeneration potential between PLLA/Aragonite and PLLA/Vaterite pearl composite scaffolds in rabbit radius segmental bone defects. Bioact. Mater..

[37] Upadhyay R.K. (2017). Role of calcium bio-minerals in regenerative medicine and tissue engineering. J. Stem Cell Res. Ther..

[38] Ma C., Jiang L., Wang Y., Gang F., Xu N., Li T., Liu Z., Chi Y., Wang X., Zhao L. (2019). 3D printing of conductive tissue engineering scaffolds containing polypyrrole nanoparticles with different morphologies and concentrations. Materials.

[39] Shuai C., Li Y., Feng P., Guo W., Yang W., Peng S. (2018). Positive feedback effects of Mg on the hydrolysis of poly-l-lactic acid (PLLA): Promoted degradation of PLLA scaffolds. Polym. Test..

[40] Yu B., Meng L., Fu S., Zhao Z., Liu Y., Wang K., Fu Q. (2018). Morphology and internal structure control over PLA microspheres by compounding PLLA and PDLA and effects on drug release behavior. Colloid. Surf. B.

[41] Wang J., Chen Q., Du B., Cao L., Lin H., Fan Z., Dong J. (2018). Enhanced bone regeneration composite scaffolds of PLLA/β-TCP matrix grafted with gelatin and Hap. Mat. Sci. Eng. C.

[42] Xiao L., Wu M., Yan F., Xie Y., Liu Z., Huang H., Yang Z., Yao S., Cai L. (2021). A radial 3D polycaprolactone nanofiber scaffold modified by biomineralization and silk fibroin coating promote bone regeneration in vivo. Int. J. Biol. Macromol..

[43] Wang J., Dai X., Peng Y., Liu M., Lu F., Yang X., Gou Z., Ye J. (2021). Digital light processing strength-strong ultra-thin bioceramic scaffolds for challengeable orbital bone regeneration and repair in Situ. Appl. Mater. Today.

[44] Eghtesad S., Nurminskaya M.V. (2013). Binding of pro-migratory serum factors to electrospun PLLA nano-fibers. J. Biomater. Sci. Polym. Ed..

[45] Piran M., Shiri M., Soufi Zomorrod M., Esmaeili E., Zomorrod M.S., Shiran N.V., Mahboudi H., Daneshpazhouh H., Dehghani N., Hosseinzadeh S. (2019). Electrospun triple-layered PLLA/gelatin. PRGF/PLLA scaffold induces fibroblast migration. J. Cell. Biochem..

[46] Zhepao Y., Huihua Y., Bingcheng Y., Xianliu W., Zhaowenbin Z., Yanzhong Z. (2018). Effects of Electrospun Fiber-Stiffness on Adhesion and Migration of iPS-MSCs. Chem. J. Chin. Univ.-Chin..

[47] Hu C., Liu S., Zhang Y., Li B., Yang H., Fan C., Cui W. (2013). Long-term drug release from electrospun fibers for in vivo inflammation prevention in the prevention of peritendinous adhesions. Acta Biomater..

[48] Dong Q.N., Kanno T., Bai Y., Sha J., Hideshima K. (2019). Bone regeneration potential of uncalcined and unsintered hydroxyapatite/poly l-lactide bioactive/osteoconductive sheet used for maxillofacial reconstructive surgery: An in vivo study. Materials.

[49] Kanno T., Sukegawa S., Karino M., Furuki Y. (2019). Navigation-assisted orbital trauma reconstruction using a bioactive osteoconductive/bioresorbable u-HA/PLLA system. J. Maxillofac. Oral Surg..

[50] Wei H., Yan S., Menary G. (2021). modelling stretch blow moulding of poly (l-lactic acid) for the manufacture of bioresorbable vascular scaffold. Polymers.

[51] Sadeghi-Avalshahr A., Khorsand-Ghayeni M., Nokhasteh S., Shahri M.M., Molavi A.M., Sadeghi-Avalshahr M. (2018). Effects of hydroxyapatite (HA) particles on the PLLA polymeric matrix for fabrication of absorbable interference screws. Polym. Bull..

[52] Ide A., Sakane M., Chen G., Shimojo H., Ushida T., Tateishi T., Wadano Y., Miyanag Y. (2001). Collagen hybridization with poly (L-lactic acid) braid promotes ligament cell migration. Mater. Sci. Eng. C.

[53] He Z., Xiong L. (2010). Evaluation of in-vitro cytotoxicity of composite materials composed of poly-L-lactic acid and β-tricalcium phosphate. Polym.-Plast. Technol. Eng..

[54] Abazari M.F., Nasiri N., Nejati F., Kohandani M., Hajati-Birgani N., Sadeghi S., Piri P., Soleimanifar F., Rezaei-Tavirani M., Mansouri V. (2021). Acceleration of osteogenic differentiation by sustained release of BMP2 in PLLA/graphene oxide nanofibrous scaffold. Polym. Adv. Technol..

[55] Bai J., Dai J., Li G. (2015). Electrospun composites of PHBV/pearl powder for bone repairing. Prog. Nat. Sci. Mater. Int..

[56] Widyowati R., Suciati S., Haryadi D.M., Chang H.-I., Suryawan I.N., Tarigan N. (2021). The effect of deer antler from East Kalimantan to increase trabecular bone density and calcium levels in serum on osteoporotic mice. J. Basic Clin. Physiol. Pharmacol..

[57] Palaveniene A., Tamburaci S., Kimna C., Glambaite K., Baniukaitiene O., Tihminlioğlu F., Liesiene J. (2019). Osteoconductive 3D porous composite scaffold from regenerated cellulose and cuttlebone-derived hydroxyapatite. J. Biomater. Appl..

[58] Zhang X., Cai Z., Li W., Zhu M. (2018). Understanding hydration effects on mechanical and impacting properties of turtle shell. J. Mech. Behav. Biomed. Mater..

[59] Chen M., Hu N., Zhou C., Lin X., Xie H., He Q. (2017). The hierarchical structure and mechanical performance of a natural nanocomposite material: The turtle shell. Colloids Surf. A Physicochem. Eng. Asp..

[60] Ma Y., Zhang C., Wang Y., Zhang L., Zhang J., Shi J., Si J., Yuan Y., Liu C. (2021). Direct three-dimensional printing of a highly customized freestanding hyperelastic bioscaffold for complex craniomaxillofacial reconstruction. Chem. Eng. J..

[61] Wysokowski M., Jesionowski T., Ehrlich H. (2018). Biosilica as a source for inspiration in biological materials science. Am. Mineral. J. Earth Planet. Mater..

[62] Yang Y., Yao Q., Pu X., Hou Z., Zhang Q. (2011). Biphasic calcium phosphate macroporous scaffolds derived from oyster shells for bone tissue engineering. Chem. Eng. J..

[63] Luo W., Zhang S., Lan Y., Huang C., Wang C., Lai X., Chen H., Ao J. (2018). 3D printed porous polycaprolactone/oyster shell powder (PCL/OSP) scaffolds for bone tissue engineering. Mat. Res. Exp..

[64] Wang Z., Han L., Sun T., Wang W., Wu B. (2021). Construction of tissue-engineered bone with differentiated osteoblasts from adipose-derived stem cell and coral scaffolds at an ectopic site. Br. J. Oral Maxillofac. Surg..

[65] Granito R.N., Custódio M.R., Rennó A.C.M. (2017). Natural marine sponges for bone tissue engineering: The state of art and future perspectives. J. Biomed. Mater. Res. Part B Appl. Biomater..

[66] Gabbai-Armelin P.R., Kido H.W., Cruz M.A., Prado J.P.S., Avanzi I.R., Custódio M.R., Renno A.C.M., Granito R.N. (2019). Characterization and cytotoxicity evaluation of a marine sponge biosilica. Mar. Biotechnol..

